# Kenyan medicinal plants used as antivenin: a comparison of plant usage

**DOI:** 10.1186/1746-4269-2-7

**Published:** 2006-02-01

**Authors:** Bethwell O Owuor, Daniel P Kisangau

**Affiliations:** 1 The Catholic University of Eastern Africa, Department of Natural Sciences, P.O. Box 62157, Nairobi, Kenya; 2 University of Dar es Salaam, Department of Botany, P.O. Box 35060, Dar es Salaam, Tanzania

## Abstract

The success of snake bite healers is vaguely understood in Kenya, partly due to their unknown materia medica and occult-mystical nature of their practice. A comparison is made of plants used in snake bite treatments by two culturally distinct African groups (the Kamba and Luo). Thirty two plants used for snakebite treatment are documented. The majority of the antidotes are prepared from freshly collected plant material – frequently leaves. Though knowledge of snake bite conditions etiological perceptions of the ethnic groups is similar, field ethnobotanical data suggests that plant species used by the two ethnic groups are independently derived. Antivenin medicinal plants effectively illustrate the cultural context of medicine. Randomness or the use of a variety of species in different families appears to be a feature of traditional snake bite treatments. A high degree of informant consensus for the species was observed. The study indicates rural Kenya inhabitants rely on medicinal plants for healthcare.

## Background

Snake bite is a major health hazard that leads to high mortality and great suffering in victims. Conservative sources estimate that the number of accidents globally reach one million, resulting in 600,000 envenomations and more than 20,000 deaths annually [1]. Other sources place annual incidences globally at 5 million with about 40,000 or more deaths – close to 10% mortality attributed to malaria [2]. In India alone more than 200,000 cases are reported and an estimated 35,000 to 50,000 people die each year [3]. A community-based retrospective survey in Kenya estimated that only 19% of the annual 151 snake bites per 100,000 people were potentially of venomous snakes [4].

Antiserum is the only therapeutic agent available throughout the world. A major drawback of serum therapy is its prohibitive cost and chance that victims are often some distance away from medical care when bitten. Serum sickness is a possible side effect of serum therapy that results in inflammation of certain tissues, and other symptoms. Generally anti-venom serum is a scarce commodity and in the world market – sometimes even governments with money to purchase large quantities cannot obtain it. There is a crisis in the quality and supply of antivenom serum in the sub-Saharan Africa due to fallen production and business pressures resulting from privatization of production plants [5].

Although it is well known that antiserum is invariably unavailable in remote rural Africa, the role of medicinal plants remains largely unnoticed and neglected. Whereas some commentators note that improvements in early referral and appropriate accident care will only occur when traditional healers are integrated into primary health care and hospital-based health systems it is important to explore their materia medica for alternative venom antidotes that could accompany or substitute conventional antivenoms. With about 250,000 species of angiosperms, this is perceptibly a rich area to explore.

Snake bite remedies are of interest since they may have recognizable therapeutic or toxic effects and are steeped in cultural beliefs that invariably conflict with formal health care practices. Ethnobotany, the study of the interaction between plants and people, is invaluable in discovering new herbal medicines and plant-derived drugs. However, glossing antiophidian plant reports for premier species in new antivenom development is a challenge in drug discovery. Studies exploring pharmacopoeia of unrelated cultures for plants treating specific medical conditions (snake bites in this study) present one way of validating anecdotal field reports, corroborating and selecting promising lead plants. This paper presents antivenin therapies from the Luo and Kamba ethnic groups of Kenya. The fact that the two communities have a fragile biomedical health delivery system, are highly exposed to snake bites yet documentation on plant remedies used as antivenin in Kenya is far less than the practice set the basis for this study. In addition the current study lays a basis for similar studies in other Kenyan communities.

Comparative studies in the two ethnic groups are made to reflect the degree of consensus of herbal remedy usage in these areas that have different agroecological zones, socio-cultural and socio-economic diversities; consensus is an indicator of the likely efficacy of the remedies in question. The immediate short-term goals of the study were aimed at conserving largely oral ethnomedical knowledge and availing to the scientific world plant therapies used as antivenin in the two communities. The long-term goal is to actualize conventional snake bite therapy options with effective, cheap, accessible and less iatrogenic (allergic) plant compounds.

## Methods

The study was carried out in rural areas of Nyanza and Eastern provinces of Kenya. Nyanza province lies astride the equator between 08359 S and 348459 E. Makueni district of the Eastern Province of Kenya lies between 135 S and 38030 E.

Like a number of World Health Organization member states, Kenya has tried to improve equity and access to health facilities. There are 60 established health facilities (hospitals, dispensaries and health centers) in Makueni district and 82 in southwestern Nyanza's Migori, Suba and Homa Bay districts [6,7]. Nevertheless the average distance patients' travel to a health center in both sites is 10–80 kilometres and this is coupled with an acute shortage of health personnel. The doctor per capita in southwestern Nyanza is 1:95734 and 1:119,879 in Makueni. These figures are far from the government's target of 1:20000. Majority of the study site populations are unable to afford biomedicine owing to poverty linked to unemployment and high medication costs [6,7]. Rainfall in the both study districts is generally scarce and varies with altitude. The incidence of malaria, gastrointestinal diseases, anaemia, pneumonia and acute respiratory diseases is fairly high in Makueni and southwestern Nyanza [6,7]. Other health problems are associated with lack of clean drinking water leading to diseases like typhoid and amoebic dysentery and poor road network for access to health facilities [6,7]. An emerging health problem, with devastating impact on agrarian livelihoods in both study sites is HIV/AIDS.

Southwestern Nyanza falls within the Lake Victoria regional mosaic plant belt of Africa, dominated by a graded vegetation landscape of relict tropical rainforest, bush grassland (*Themeda*-*Hyparrheni*a) and wooded grassland vegetation of the *Combret*o-*Dodonea*e-*Balanite*s-*Acacia *matrix. Makueni district is in the Somalia – Maasai phytochorion – an area occupied largely by wooded, dry bushland and grassland vegetation with *Commiphora*-*Acacia*-*Combretum *communities [8]. Southwestern Nyanza (Migori, Homa Bay and Suba district) is predominantly occupied by the Luo tribe who speak *Dholuo *and Makueni district by the Kamba tribe who speak *Kikamba*. The Luo had their original home somewhere in Southern Sudan [9]. The Kamba migrated eastward into Kenya from Central Africa. The Luo people of Nyanza province belong to the Western Nilotic cluster of societies and their language has a Nilo-Saharan eastern Sudanic affiliation. The Kamba people of Eastern province belong to the Central-Kenya Group of Bantu-speaking people of the Niger-Congo language family. The Kamba language is a cluster of four dialects; South Kitui, Masaku, North Kitui and Mumoni. Both communities are patrilineal and patrilocal and invariably polygynous. Though both communities are sedentary, the Luo were in the past majorly pastoral but practiced limited cultivation. The Kamba supplemented their agricultural and semi-nomadic economy, in times of food shortage e.g during famine, with a barter trade called *'kuthuua'*. Adults sought out for food in Kamba territory and unrelated neighbouring peoples by offering labour, buying or bartering.

Both studies were conducted in broad ethnobotanical studies interviewing laypersons and specialist traditional practitioners over an eleven-month period in 1998 and 1999. Knowledgeable members of the society were chosen with aid of local community members and local administration. Identification of authentic practitioners was done in a manner similar to that described in [10]. Local community members were requested to list all known herbalists in their neighbourhood and carry out pooled comparison from the list, and rank the practitioners according to the community's confidence on each practitioner. One hundred and twelve respondents were interviewed in the Kamba study and one hundred in southwestern Nyanza. Of these 112 respondents, 19 were highly reputed herbal practitioners – 12 men and 7 women. The sixteen specialist traditional healers interviewed, out of one hundred respondents, in southwestern Nyanza ranged from 32–63 years of age and 40–75 years in the Kamba study.

With the help of field research assistants, the researchers were involved in administering questionnaires during in-depth interviews. There were however pre-arranged appointments (like 2–3 days before) with the respondents' through/with the help of local administrators. Healers were asked to state plant names and their cultural meanings, medicine preparation methods and symptoms of deleterious snakebites. The research procedure was reviewed and approved by the Graduate Research Committee of the Department of Botany, Faculty of Science of the University of Nairobi. Clearance to conduct research was obtained from local Government of Kenya administrative offices (Chiefs and District officers) before the fieldwork started.

Prior informed consent of informants was sought before questionnaires with both close and open-ended questions as in [11] were used to collect survey data. The interviews were conducted in the local *Dholuo *and *Kikamba *language. Data collection entailed re-turn visits and transect walks in which samples of the herbal medicines were observed and collected. Voucher specimens of medicinal plants, in triplicates, were collected, prepared and identified. They were later verified before deposition at the University of Nairobi herbarium. After the interview tokens of appreciation, in good form, were given as appreciation of interviewee time. The study was also facilitated by available corpus of literature focussing on ethnobotany of medicinal plants used by the Kamba and Luo. Factor of informant consensus (F_ic_) adopted from [12] is used in analyzing and comparing snake bite remedies of Kamba and Luo traditions.

## Results

The research respondents were peasant farmers, pastor, adult school teachers, diviners, full time herbalists, masons and carpenters. In terms of education: 44% of the research informants had no formal education, 10% high school; 40% primary education and below; and 4% adult education. The information in table 1 is for the total 212 respondents interviewed. In general the more educated respondents had less knowledge on these remedies.

**Table 1:** Age range of the Kamba and Luo informants

**Age range (Years)**	**21–35**	**21–35**	**36–50**	**36–50**	**51–65**	**51–65**	**66–80**	**66–80**	**>80**	**>80**	**Total**	**Total**
Location	**MAK**	**MIG**	**MAK**	**MIG**	**MAK**	**MIG**	**MAK**	**MIG**	**MAK**	**MIG**	**MAK**	**MIG**

Males	3	18	9	23	21	13	26	8	4	1	**63**	**64**
Females	0	4	28	7	6	8	13	12	2	6	**49**	**36**
Totals											**112**	**100**

KEY: MAK – Makueni, MIG – Migori

### Perceptions of snakes and snakebites

Snakes are called '*nzoka*' and *'thuol' *in the Kamba and Luo language respectively. In Kambaland, a number of venomous species were identified: *"nzoka ya kiko" *or *Naja melanoleuca *(Fig. 2); the very fierce *"yaitha" *or *Dendroaspis polylepis *and *'kimbuva'*" or *Bitis *spp. (Fig. 3) the puff adders. With exception of the spitting cobra, these snakes typically deliver venom through two hollow teeth called fangs; the venom is called *'kiri' in Dholuo *and *"sumu" *in *Kikamba*. Spitting cobras are notable for visiting settled areas and are reputed to spit venom toward enemies up to 2.5 m away. There was concurrence among the informants that some snake bite cases were caused by malevolent persons.

**Figure 1:**
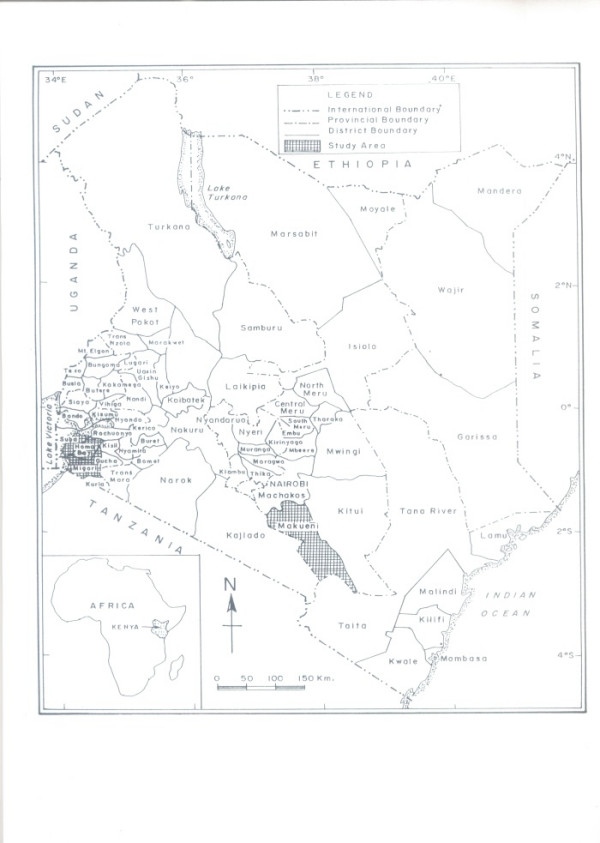
Map of Kenya showing study sites.

**Figure 2:**
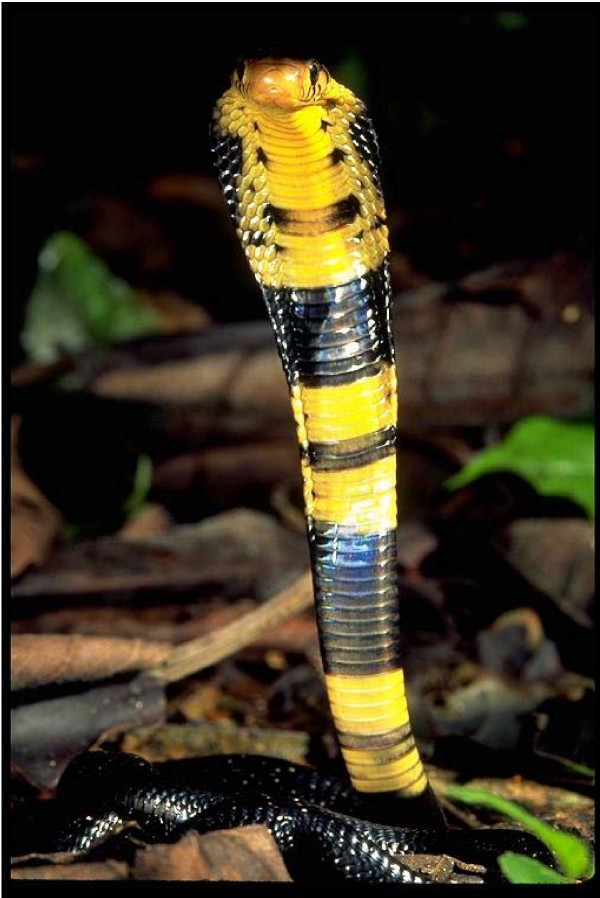
Naja melanoleuca.

**Figure 3:**
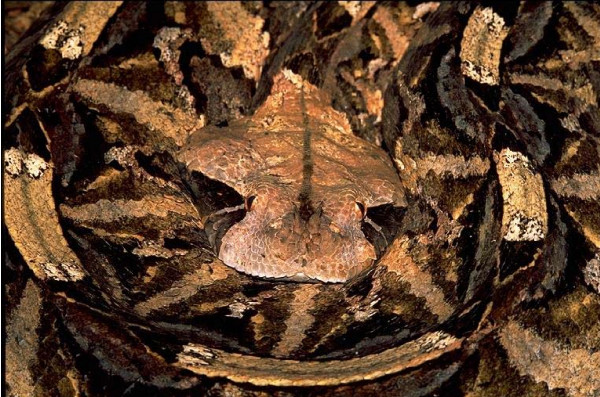
Bitis gabonica.

Snake bites were considered a matter of emergency in both study sites. Venomous bites usually show as double teeth marks. Systemic manifestations of envenomation included generalized weakness, nausea, and fever and shock. Snake bites are common during the hot and dry season and around the rainy season. The local explanation was that snakes move around looking for water during the dry seasons. Herdsmen, cultivators and firewood collectors are very prone to bites. Informants from both ethnicities noted the most poisonous snakebites are those of *Bitis *spp. In the event of envenomation accidents, victims were reassured and barred from physical exertion. Incisions are made around the bite and herbal remedies applied on the wound site. Antidotes are administered not later than half an hour after the bite.

### Snake bite healers

The traditional healers are the first line defence against illnesses. The success of these healers is vaguely understood, partly due to their unknown *materia medica *and occult-mystical nature of their practice, but direct testimony from victims confirms success of their treatments. Biomedicine ignores their practice but they serve more snake bite accident victims than modern practitioners. The healers, especially the elderly and spirit inspired, are reputed to have an inherent expertise to handle these cases. In both societies they are recognized as healthcare providers with a profound socio-cultural understanding of their communities. They offer a blend of solace, advice and therapy usually delivered within an understanding of the patient's background.

The Kamba traditional healers, *"andu awe" *(plural) *"mundu muwe" *(singular) are inducted into healing through apprenticeships and some inherit lineal practice by possession with ancestral spirits *"maimu"*-such healers invariably have inspiration and contact with these spirits through dreams. They have spiritual herbal skills and invoke spiritual healings. Among the Kamba there are some herbal practitioners who are Christians and pray before administering herbs. A similarly practice is found in Luo prayer specialists or *'jolemo'*. Persons knowledgeable in these antidotes owe their healing skills to training by knowledgeable kin, spirit inspiration, or *'nyiewo yath' *(buying medical skill from a non-relative specialist). Many, more than 60%, of the healers interviewed in Makueni were elderly and lacked apprentices. We observe that as the snake bite practitioners die, much of the knowledge they possess dies too. In terms of professional experience most of the informants (39%) had little professional experience i.e. 1–5 years. Only 19% had experience exceeding 21 years and most in this category were full time healers. Over millennia Kamba and Luo ethnomedical knowledge was conveyed orally to younger generations; however over the last two decades transmission gaps have developed largely because of changed lifestyle and focus on a formal type of education system that does not offer informal traditional knowledge.

### Herbal remedies

In Makueni herbs used in treating these accidents are *'miti ya kuiita kuumwa ni nzoka' *or snake bite herbs. Similar remedies are called *'yadh thuol' *literally meaning snake medicine in southwestern Nyanza. The Luo remedies are after Owuor *et al*. 2005 [13]. Most species in table 2 are indigenous, however, both communities had two exotic species *Allium cepa *and *Tagetes minuta *(Kamba) and *Senna siamea *and *Tithonia diversifolia *among the Luo of southwestern Kenya. These plants were prepared as infusions; decoctions or macerations. In some treatments, the snake teeth were mixed with *Opilia amentecea*, a woody vine, to treat snake bite poisoning. Among the Luo some therapy involved oral consumption of egg yolk and albumin before treatment.

**Table 2:** Plants used as antivenins

**Plant**	**Family**	**Local names**	**Preparation/specimen no.**	**Remedy reports**
*Allium cepa *L.	Iridaceae	*Kitunguu *(Kamba name)	Leaves or root tuber pounded and sap applied. KD 422 (NAI).	2
*Ammocharis tinneana *(Kotschy & Peyr.) Milne-Redh.	Amaryllidaceae	*Apap thwon pap, rabwond otenga*****(Luo name)	Root sap used in the preparation of a snake bite alexiteric. BOO 250 (NAI).	1
*Annona senegalensis *Pers. ssp. *senegalensis*	Annonaceae	*Obolo, obolobolo*****(Luo name)	The crushed leaves are rubbed on snake bites, some chewed and the juice swallowed. BOO 456 (NAI).	3
*Bidens pilosa *L.	Asteraceae	*Nyanyiek mon, onyiego*****(Luo name)	Crushed leaves of the plant are rubbed on fresh cuts as an astringent, snake bite antidote and antiseptic. BOO 485 (NAI).	4
*Senna siamea *(Lam.) Irwin et Barnaby	Fabaceae	*Ndege owinu, oyieko*****(Luo name)	The roots of this tree and those of *Zanthoxylum chalybeum *are used as antidote for snake bites. BOO 401 (NAI).	1
*Combretum collinum *Fresen.	Combretaceae	*Adugo*****(Luo name)	The roots are used in preparing a snake bite antidote that is usually effected by scarification. BOO 647 (NAI).	3
*Combretum molle *G. Don	Combretaceae	*Muama, Kiama *(Kamba name)	Root or bark pounded, soaked in water and infusion drunk; 2 glasses two times a day. KD501 (NAI).	9
*Conyza sumatrensis *(Retz.) E. Walker.	Asteraceae	*Yadh asere, yadh tong*' (Luo name)	A leaf infusion of the plant is drunk as an antidote for puff adder (*Bitis arietans*) bites and stomachache. BOO 534 (NAI).	7
*Corchurus trilocularis *L.	Tiliaceae	*Apoth*****(Luo name)	The crushed leaf infusion with *Hyptis pectinata *is dropped or sprinkled into the eye to neutralize snake venom ejected into the human eye. Afterwards, the victim is scarified on the hind torso. Claims of intense pain, temporary blindness and watery eyes were linked to this type of venom poisoning. BOO 559 (NAI).	1
*Dichondra repens *J.R. Forst. & G. Forst.	Convolvulaceae	No Luo name given	The plant leaves are rubbed onto snakebites to as an antidote to "remove snake fangs". BOO 536 (NAI).	1
*Ensete edule *(J. F. Gmel.) Horan	Musaceae	*Kitembe*****(Luo name)	The sap exuding from cut stem is used in treating snake bites, wiped into the bite. BOO 181 (NAI).	1
*Entada leptostachya *Harms	Fabaceae	*Mwaitha *(Kamba name)	Stem crushed, sap squeezed out and applied. KD 458 (NAI).	7
*Erythrina excelsa *Baker	Fabaceae	*Roko, yuoma*****(Luo name)	The bark sap is antidote for snake bites. BOO 691 (NAI).	2
*Fuerstia africana *T.C.E. Fr.	Lamiaceae	*Abunga-useke, aremo*****(Luo name)	Leaves are crushed and the filtered infusion prepared thereafter drank orally as antidote. BOO 523 (NAI).	1
*Grewia *sp.	Tiliaceae	*Powo*****(Luo name)	The leaves are snake bite antidote. Leaves used in cooking envenomed carcass – as a treatment preventing secondary poisoning. Livestock bitten by snakes are drenched with a leaf**/ **bark decoction/infusion and the mucilaginous crushed leaves used to wipe the bitten area. BOO 248 (NAI).	3
*Indigofera circinella *Baker f.	Fabaceae	*Odolo*****(Luo name)	Poultices made from the leaves are chewed and pasted on snake bite as antidote. BOO 566 (NAI).	3
*Justicia calyculata *(Deflers.) T. Anders.	Acanthaceae	*Apiwo, piu piu*****(Luo name)	Crushed aerial plant parts are used as a snake bite antidote, rubbed onto the snake bite to facilitate the removal of the snake's fangs. BOO 486 (NAI).	1
*Laggera brevipes *Oliv. & Hiern.	Asteraceae	*Adupa rabuor*****(Luo name)	The roots of the plant are employed as a snake bite antidote. BOO 304 (NAI).	3
*Maesa lanceolata *Forssk.	Myricaceae	*Katera*****(Luo name)	Root decoction administered as follow up treatment for puff adder (*Bitis *spp.) bites. BOO 292 (NAI).	2
*Microglossa pyrifolia *(Lam.) Kuntze	Asteraceae	*Nyabung odidi, nyabung odit*****(Luo name)	The leaves are chewed, juice swallowed and the macerate placed well into the snake bite. BOO 463 (NAI).	5
*Opilia amentacea *Roxb.	Opiliaceae	*Mutonga *(Kamba name)	Roots burnt into charcoal, crushed into powder mixed with crushed snake teeth and applied to treat the snake bites. KD 503 (NAI).	16
*Pellaea viridis *(Forssk.) Prantl	Adiantaceae	No Luo name given	The plant leaves are pulped and rubbed well into a snake bite. BOO 570 (NAI).	1
*Sansevieria parva *N.E.Br.	Dracaenaceae	*Twoch bungu*****(Luo name)	Leaf sap applied on snake bite wound. BOO 127 (NAI).	1
*Solanecio mannii *(Hook. f.) C. Jeffrey	Asteraceae	*Maroo, marowo*****(Luo name)	Crushed or chewed leaves rubbed into snake bites as antidote. BOO 681 (NAI).	2
Solanum incanum L.	Solanaceae	*Mutongu *(Kamba name)	The stem or fruits cut into small pieces, dried in sun, pounded and powder applied. The sap of the fruits may also be directly applied. KD 393 (NAI).	9
*Steganotaenia araliacea *Hochst.	Apiaceae	*Muvuavui, Kivuavui *(Kamba name)	Roots burnt into charcoal, crushed into powder and applied. KD 509 (NAI).	2
*Tagetes minuta *L.	Asteraceae	*Muvangi *(Kamba name)	Leaves crushed, soaked in water and infusion applied. KD 468 (NAI).	5
*Tithonia diversifolia *(Hemsl.) A. Gray.	Asteraceae	*Maua madongo, akech*****(Luo name)	Leaf infusion administered orally as antidote for snake bites. BOO 541 (NAI).	3
*Triumfetta rhomboidea *Jacq.	Tiliaceae	*Muinda nguue *(Kamba name)	Roots crushed, soaked in water and infusion applied on bite area. KD 519 (NAI).	3
*Uvaria scheffleri *Diels	Annonaceae	*Mukukuma *(Kamba name)	Roots or leaves dried in sun and pounded and powder applied. KD 324 (NAI).	4
*Vernonia glabra *(Steez) Vatke	Asteraceae	*Olusia*****(Luo name)	The leaf ash or crushed leaves rubbed into scarifications around the snake bite as antidote. BOO 759 (NAI).	1

### Parallel use reports

The linkages between plant diversity and human cultural diversity can be understood in cross-cultural study of plant usage, both as symbols (in art and ritual) and as materials (in food, medicine, construction and handicrafts). Linkage of Kamba and Luo remedy choice point to potential in developing new snakebite remedies. A basic test for cultural analysis is whether both culture groups use similar plants. Our results, though with observable similarities in plant families used as antivenins, do not show a clear association of Kamba and Luo remedies. Despite this difference the Luo and Kamba beliefs of snake bite perception and etiology link. Only the genus *Combretum *is shared amongst the two ethnic groups. Similar usage of *Combretum *species is reported in Tanzania [14]. A further possible indicator of efficacy likelihood in the family Combretaceae is observed by usage of *Terminalia superba *[15]. The observed differences in species used by the groups strengthens the implication of culture and ecology in antivenin choice and an assumption that randomness or the use of a variety of species in different families is a feature traditional snake bite treatments.

The use of differing species invites discussion about the selection of medicinal plant remedies for snake bite. To further evaluate the variability of the use of medicinal plants and to determine whether pharmacopoeia from a particular ethnic group is of interest in the search for bioactive compounds, our analysis in table 3 draws upon the informant consensus factor (F_ic_) [12]. F_ic _gives the relationship between the "number of use-reports in each category (n_ur_) and number of taxa used (n_t_)".

**Table 3:** Consensus of antivenin remedies

	**Kamba**	**Luo**
Total number of taxa (n_t_)	8	24
Number of plant families	8	13
Total number of usage reports (n_ur_)	57	50
Informant consensus (F_ic_)	0.875	0.53

F_ic _= (n_ur _- n_t_/n_ur _- 1)

They compared the total case-number for each ailment (number of informants that reported a certain illness) with the number of separate remedies for this ailment.

A high F_ic _value (close to 1) indicates that the informants (lay persons and herbalists) use relatively few taxa, while a low value indicates that the informants disagree on the taxa to be used in the treatment within a category of illness. The greater the consensus factor the more likely it is that the remedy has bioactive molecules. F_ic _for snake bite remedies is higher among the Kamba – it means there is a high degree of informant consensus for remedies used by the Kamba informants. This implies Kamba remedies are more consistent, yet in terms of family and species diversity the results from the Luo informants are richer. In a study among the Baka of Cameroon 2.7% (28 reports) of the total 1037 reports are attributed to usage of 9 plant antivenin taxa [15]. This translates into a F_ic _value of 0.70, a figure comparing favourably with the Kamba F_ic_. The low number of usage reports among the Baka possibly points to fewer snake bite incidences relative to Kenya.

## Discussion

Snake bites in rural areas are commonly treated with plant extracts [16-21]. Corresponding usage reports are found for *Steganotaenia araliacea*, *Combretum collinum*, *Solanum incanum *and 3 species of *Grewia *(*G. bicolor*, *G. fallax *and *G. truncata*) in the *Medicinal Plants of East Africa *[22]. In addition species in the genera *Vernonia*, *Erythrina (E. abyssinica) and Sansevieria *(*S. kirkii*) are recorded. There are similar usage reports for *Annona *(leaf and bark) and among the Chewa ethnic group of Malawi *Ensete edule *(sap from stem) as a toothache pain reliever – alluding to painkilling properties; arterenol, a natural mediator of the autonomic nervous system, is reported in *E. edule *[23]. Usage of *Allium *sp., in South America, as antivenom is reported [24]. *Allium cepa *contain sulfurous, volatile oils [25].

Though in general the plant families Compositae, Leguminosae and Solanaceae and Apocynaceae are well represented in East African compendia [22,23] it appears plant families or genera consistently used in snake bite treatment are difficult to establish. The plant family Asteraceae leads and is followed by Annonaceae, Fabaceae, Combretaceae and Tiliaceae in this study. Among the Baka nearly all remedies are from Apocynaceae and Annonaceae [15]. This observation requires further documentation and collation of ethnobotanical results.

The frequent usage of leaves and roots in antivenin preparations is noted in [22,23]. Most of the indigenous remedies reported in literature are root-derived. Various plants named "snakeroot" with long twisted "snaking" roots are preferred. In North America and Asia, this name applies to at least 5 different plants supposed to be snake bite remedies [26].

Traditional healers have reputation of treating difficult snake bite cases and are trusted by their patients. In both study areas, cases of deaths in victims attended by traditional healers were very rare, (less than 3%). In a Colombian study healers interviewed reported only 4.4% death in cases they handled [17].

Though medicinal plants remain largely unnoticed and neglected, protective activity of plant extracts have been confirmed in biological assays: resverotrol (3,4'5-trihydroxy trans-stilbene) from a snake bite herbal *Cissus assamica*, [20]; reduction of venom-induced effects of *N. nigricollis *in rats by pre-incubation with *Parkia biglobosa *extracts [19]; and activation of coagulative (prothrombin) activity by *Mucuna pruriens *seed extract [27]. Anti-inflammatory activity in *B. pilosa *is recorded [28,29].

Pure substances from plants shown to protect mice from ophitoxaemia are generally nitrogen-free, low-molecular-weight compounds: phenolics, phytosterols (β-amyrin and sitosterol) and triterpenoids [30-34] but exceptions are found in 12-methoxy-4-methylvoachalotine, an alkaloid [35]. Proposed views advanced in [30-36] indicate that these micromolecules interact with macromolecular targets; receptors and enzymes; resulting in venom-inactivation, analgesic and anti-inflammatory action [37].

## Conclusion

The Luo and Kamba communities have used natural medicines from plants for generations. This usage is influenced by the existence of an inadequate biomedical health system, cost-effectiveness and cultural acceptability of plant-based therapies. Exceptionally skilled ubiquitous healers and individuals in these societies administer the remedies. Generally the consensus among users indicates these plants have protective activity when administered in snake bite situations. Our results show that plant species used by the two ethnic groups are independently derived. The variation of antivenin medicinal plants in these communities effectively illustrates the cultural context of medicine. Each community's remedy choice is coloured by their cultural background.

## Competing interests

The author(s) declare that they have no competing interests.

## Authors' contributions

OB and KD executed the ethnobotanical surveys and identified plant material described.
